# Radix Astragali-Based Chinese Herbal Medicine for Oxaliplatin-Induced Peripheral Neuropathy: A Systematic Review and Meta-Analysis

**DOI:** 10.1155/2016/2421876

**Published:** 2016-10-04

**Authors:** Bo Deng, Liqun Jia, Zhiqiang Cheng

**Affiliations:** Department of Oncology of Integrative Chinese and Western Medicine, China-Japan Friendship Hospital, Beijing 100029, China

## Abstract

*Background*. Treatment of chemotherapy-induced peripheral neuropathy (CIPN) remains a big challenge for oncologists. The aim of this study is to evaluate the effects of Radix Astragali- (RA-) based Chinese herbal medicine in the prevention and treatment of oxaliplatin-induced peripheral neuropathy, including the incidence and grading of neurotoxicity, effective percentage, and nerve conduction velocity.* Methods*. All randomized controlled trials (RCTs) were found using PubMed, Cochrane, Springer, China National Knowledge Infrastructure (CNKI), and Wanfang Database of China Science Periodical Database (CSPD) by keyword search. Meta-analysis was conducted using RevMan 5.0.* Results*. A total of 1552 participants were included in 24 trials. Meta-analysis showed the incidence of all-grade neurotoxicity was significantly lower in experimental groups and high-grade neurotoxicity was also significantly less. Effective percentage was significantly higher and sensory nerve conduction velocity was improved significantly, but changes in motor nerve conduction velocity were not statistically significant. No adverse events associated with RA-based intervention were reported.* Conclusion*. RA-based intervention may be beneficial in relieving oxaliplatin-induced peripheral neuropathy. However, more double-blind, multicenter, large-scale RCTs are needed to support this theory.* Trial Registration*. PROSPERO International prospective register of systematic reviews has registration number  CRD42015019903.

## 1. Introduction

Chemotherapy-induced peripheral neuropathy (CIPN) results from toxic effects of chemotherapy drugs predominantly affecting the peripheral nervous system. The associated pain of CIPN can be extremely disabling, with a marked impact on quality of life (Qol), functions of daily living, and increases the risks of noncompliance with cancer treatment [1]. Oxaliplatin (OXAL), a third-generation platinum-based compound, has become pivotal for the therapy of metastatic colorectal cancer and other malignancies including lung, breast, and ovarian cancers [2, 3]. However, OXAL induced chronic neurotoxicity occurs in 63.6% or more of patients, which limited the dosing of OXAL [4].

Radix Astragali (the root of* Astragalus mongholicus Bge. *or* Astragalus membranaceus Bge.*) has been used as one of the primary tonic herbs in traditional Chinese and Japanese Kampo medicine. Recently, Radix Astragali (Huangqi, in Chinese) is being widely used, orally or topically, and alone or in combination with western conventional medicine to relieve CIPN. Multiple randomized clinical trials have suggested that Radix Astragali- (RA-) based intervention can reduce symptoms, improve Qol and immunologic function, increase plasma nerve growth factor (NGF) levels, and delay the progression of CIPN [5–28].* In vivo* RA-based prescription (Huangqi Guizhi Wuwu Decoction) can effectively relieve pain and improve sciatic nerve conduction velocity and function in rats with CIPN [29, 30]. Its mechanism may be related to downregulating NR2B expression in L4–6 lumbar spinal segments and upregulating pNF-H protein levels in dorsal root ganglia [30]. However, no systematic review to date has reported effects of RA-based intervention on OXAL induced peripheral neuropathy. In this meta-analysis, the effectiveness and safety of RA-based intervention for preventing and treating OXAL induced peripheral neuropathy are evaluated for the first time.

## 2. Methods

Ethics data for this study were acquired through previously published work; no patient or hospital data were accessed. Therefore, written consent and institutional ethical review were not required for this research.

### 2.1. Database and Search Strategies

The electronic databases of MEDLINE (1982–2015), Cochrane Controlled Trials (2015, Issue 12), Springer (1997–2015), China National Knowledge Infrastructure (CNKI) database (1997–2015), and Wanfang Database of China Science Periodical Database (CSPD) (1998–2015) were searched by using keywords of “Neurotoxicity”, “Oxaliplatin” “Astragali”, or “Huangqi”, without language limitation. Reference lists from trials selected by electronic searching were hand searched. All of those searches ended before January 2016.

### 2.2. Inclusion Criteria

All randomized controlled trials (RCTs) investigating the effects of RA-based Chinese herbal medicine for preventing and treating OXAL induced peripheral neuropathy will be eligible for inclusion.

#### 2.2.1. Types of Participants

All adult patients (18 years and older, no upper age limit) with a treatment of OXAL will be considered for this review. The participants had to conform to the following diagnostic criteria.

1. The patient was clearly diagnosed malignant by pathology or cytology.
2. The patient was treated by OXAL, FOLFOX (OXAL + 5-fluorouracil + calcium folinate), or XELOX (OXAL + capecitabine).
3. Age, gender, stages, and pathological types between the groups were balanced and comparable.

#### 2.2.2. Types of Interventions

RA-based interventions included single herb (including extracts from RA) and a compound of several herbs, irrespective of dosage form (e.g., oral decoction or lotion). The mode of delivery (e.g., oral, topical administration or intravenous) was not restricted. Relative high dose RA (monarch drug) should be included in the prescription and regimen of herbs was not restricted.

The control interventions were placebo, no intervention, or conventional treatment such as mecobalamin, Ca/Mg infusions, or reduced glutathione. We also included trials of RA-based prescription plus conventional medicine versus the same conventional medicine alone.

### 2.3. Types of Outcome Measures

*Grading of CIPN*. Primary outcome was the grading of CIPN in at least 1 chemotherapy cycle, but preferably in 4 cycles of chemotherapy. We considered Levi's grade [31], World Health Organization (WHO) grade [32] or National Cancer Institute common terminology criteria for adverse events (NCI-CTCAE) for the clinical grading of CIPN [33] (Table 1).

*Clinical Effectiveness*. Clinical effectiveness was assessed according to what is previously described [32, 34]. 

*Complete Remission (CR)*. The patients felt completely free from all symptoms, with the grading of CIPN reduced to grade 0.

*Partial Remission (PR)*. Symptoms abated obviously, and the grading of CIPN reduced ≥1 grade. 

*Nonperceptible (NP)*. Compared with before treatment symptoms have not abated, and the grading of CIPN did not reduce.

(1) $$\begin{array}{c} Remission\;\;Rate=CR+PR. \end{array}$$

*Nerve Conduction Velocity*. Changes in values of sensory nerve conduction velocity (SNCV) or motor sensory nerve conduction velocity (MNCV) were measured by validated methods after 1 week of RA treatment or more.

*Quality of Life (Qol) and Adverse Events*. We extracted Qol, measured as Karnofsky (KPS) scale or Eastern Cooperative Oncology Group (ECOG) scale. Adverse events were also extracted.

### 2.4. Exclusion Criteria

We excluded studies with unclear diagnostic criteria and without the use of RA. Combinations of herbs and other forms of treatment (e.g., acupuncture or moxibustion) were excluded.

### 2.5. Data Extraction and Quality Assessment

Data were entered into an electronic database by two authors (Bo Deng and Liqun Jia) independently. Where differences in opinion existed, they were resolved by a third party. Improved Jadad scale was used to assess the quality of RCTs, including randomization, blinding of participants, personnel, and outcome assessors, incomplete outcome data, and other threats to validity [35]. High quality is 4–7 points. Low quality is 1–3 points.

### 2.6. Data Synthesis

Review Manager (RevMan) 5.0 software, provided by the Cochrane Collaboration (UK), was used to analyze the results of the trials. Dichotomous data were expressed as odds ratio (OR). Continuous data were expressed as mean difference (MD). Heterogeneity between results of different trials was tested, and heterogeneity was presented as significant when *I* ^2^ is over 50% or *P* < 0.1. Random effect model was used for the meta-analysis if there was significant heterogeneity and fixed effect model was used when the heterogeneity was not significant [35]. Publication bias was explored via a funnel plot analysis.

## 3. Results

### 3.1. Description of Studies and Methodological Quality (Figure 1 and Table 2)

Our primary searches identified 841 references from the above databases. After duplicates, animal studies, case reports, reviews, and obvious ineligibility were removed, we retrieved a total of 110 references for further assessment. After full-text reviews, 24 trials were included [5–28]. Included trials were published from 2009 to 2015, with the years 2011 to 2015 having a larger number of trials (20 trials, 85.70% patients) than other years. All trials were conducted in mainland China. Since all included trials were assessed to be of high quality (improved Jadad score of 4 or 5 points), the risk of bias in this systematic review was low. All 24 trials employed computer software or random number tables for randomization. Nine trials used conventional medicine as control, and only one trial performed double-blinding.

### 3.2. Participants

In total, 1552 participants with OXAL treatment were included in these 24 trials. The average size of the trials was 66 participants, ranging from 40 to 135 per trial. Eleven trials enrolled only inpatients (*n* = 689 patients, 44.39%). The remaining 13 trials did not specify the setting (*n* = 863 patients, 55.61%). All trials included both adult male and female patients, with 58.63% participants being male. Types of cancer in participants included colorectal cancer (*n* = 1033 patients), gastric cancer (*n* = 399 patients), and lung cancer/breast cancer/other cancers (*n* = 52 patients). The cancers of 68 patients were not specified. Accumulated OXAL dose varied from 130 mg/m^2^ to 800 mg/m^2^, with 260–600 mg/m^2^ (11 trials) being the most common. Eighteen trials used Levi's grading of CIPN, 3 used CTCAE criteria of CIPN, and 3 used WHO criteria of CIPN.

### 3.3. Intervention Comparisons (Tables 3 and 4)

Sixteen trials (*n* = 1060 patients) compared RA-based intervention with no intervention. Three trials (*n* = 159 patients) tested RA-based prescriptions against mecobalamin. Another 5 trials (*n* = 333 patients) tested RA-based prescriptions in combination treatment remedies compared to the same western medications for CIPN management. Three types of administration methods were employed in these 24 trials, including oral administration (10 trials), topical administration (12 trials), and intravenous drip (1 trial). One trial employed oral administration combined with topical administration. The most popular prescriptions were modified Huangqi Guizhi Wuwu Decoction (7 trials) and modified Buyang Huanwu Decoction (5 trials). Prescriptions composed by the investigators themselves were combined and modified from these 2 prescriptions (10 trials). More than 50% of RA-based prescriptions included Danggui, Guizhi, Baishao, Jixueteng, Chuanxiong, and Honghua. These herbs may augment the effects of RA intervention on CIPN. Doses of RA ranged from 15 g to 180 g but most fell in the range of 30 to 50 g (12 trials). The duration of treatment varied mostly from 2 weeks to 8 chemotherapy cycles. Regarding topical administration, the temperature of decoction ranged from 35°C to 42°C, but most were in the range of 38–42°C (6 trials).

### 3.4. Effects of Interventions

#### 3.4.1. Incidence of All-Grade CIPN (Figure 2)

Eighteen trials reported incidence of all-grade (grades 1–4) CIPN. Five trials included CIPN patients and reported curative effects of RA-based prescriptions. And 1 trial only reported incidence of high-grade CIPN. Fifteen trials compared RA-based intervention to no intervention. RA-based intervention significantly reduced CIPN occurrence (*n* = 993 patients; OR, 0.19, 95% CI, 0.14 to 0.25, *P* < 0.01). One trial compared RA-based prescription to mecobalamin. RA-based prescription significantly reduced CIPN occurrence (*n* = 42 patients; OR, 0.17, 95% CI, 0.03 to 0.94, *P* < 0.05). Two trials compared RA-based prescriptions plus reduced glutathione or Ca/Mg infusions with the same conventional medications. RA-based prescriptions in combined remedies significantly reduced CIPN occurrence (*n* = 120 patients; OR, 0.42, 95% CI, 0.18 to 0.97, *P* < 0.05).

#### 3.4.2. Incidence of High-Grade CIPN (Figure 3)

Nineteen trials reported incidence of high-grade (grades 3-4) CIPN. No patients develop high-grade CIPN in 1 trial. Therefore 18 trials were included in a forest plot. Fourteen trials compared RA-based intervention to no intervention, mostly by using Levi's grading (11 trials). RA-based intervention significantly reduced high-grade CIPN (*n* = 931 patients; OR, 0.17, 95% CI, 0.09 to 0.31, *P* < 0.01). However, 2 trials compared modified RA-based prescriptions to mecobalamin, and 2 trials compared RA-based prescriptions plus reduced glutathione or Ca/Mg infusions with the same conventional medications. In these trials, there was no statistical difference between groups.

#### 3.4.3. Curative Effect of RA-Based Prescriptions (Figure 4)

Five trials included 341 patients that had already developed CIPN and reported curative effects of RA-based prescriptions. The total effective rate of RA-based prescriptions was 79.07%, compared with 54.44% in the control group. Three trials compared curative effects of RA-based prescriptions plus mecobalamin to mecobalamin alone, where RA-based prescriptions were significantly more effective in relieving CIPN (*n* = 213 patients; OR, 4.84, 95% CI, 2.38 to 9.83, *P* < 0.01). However, 1 trial compared RA-based prescription to mecobalamin, and 1 trial compared RA-based prescription to no treatment. In these trials, there was no statistical difference between groups.

#### 3.4.4. SNCV and MNCV (Figures 5 and 6)

Six trials reported RA-based interventions significantly improved SNCV (MD 4.42 m/s, 95% CI 3.27 to 5.57, *P* < 0.01). However, regarding MNCV, there was no statistical difference between groups.

#### 3.4.5. Safety, Quality of Life, and Publication Bias

**Figure 7:**
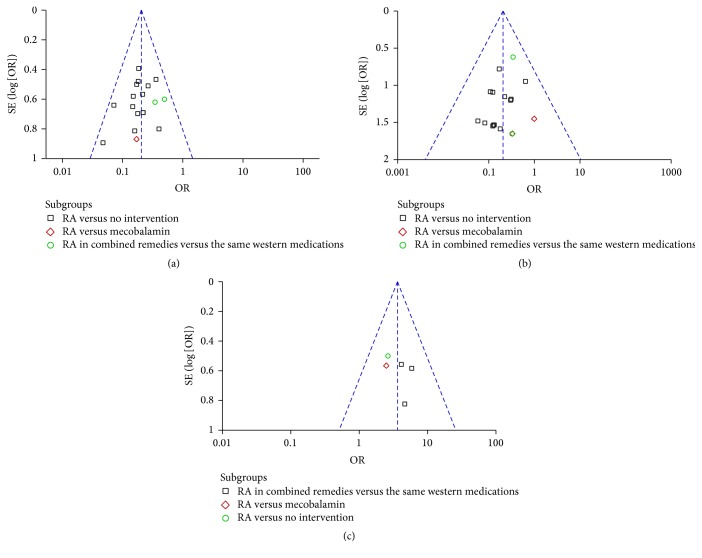
Funnel plot analysis of risk of bias. (a) Funnel plot analysis of incidence of all-grade chemotherapy-induced peripheral neuropathy (CIPN). (b) Funnel plot analysis of incidence high-grade CIPN (grades 3-4). (c) Funnel plot analysis of curative effects of Radix Astragali-based prescriptions on CIPN.

Among the 24 articles incorporated in the meta-analysis, no adverse events associated with RA-based interventions were reported. Nineteen trials reported Qol (KPS score > 60 or ECOG score ≤ 2) before RA intervention, and 2 trials reported Qol improvement. One trial reported the percentage of patients with Qol improvement while the other reported the increased level of KPS score. Therefore, the results of these 2 trials could not be combined in the meta-analysis. Exploration of the funnel plots (Figure 7) for all-grade CIPN, high-grade CIPN, and curative effects between RA-based interventions and control suggested near symmetry. No significant publication bias was found.

## 4. Discussion 

CIPN is not recorded in classic TCM books, so it remains a big challenge for TCM oncologists. Based on syndrome differentiation and treatment, TCM oncologists believe that CIPN falls under the category of Bi syndrome in TCM. The pathogenesis of CIPN is believed to be asthenia of qi and blood, qi stagnation and blood stasis. These lead to tendon and vessel malnutrition and stasis in collaterals. The treatment includes benefiting qi and nourishing blood, regulating ying and wei, and promoting blood circulation to remove meridian obstruction.

RA is one of the most commonly used herbs tonifying qi.* In vitro* and* in vivo* studies suggest RA extract can be a potential nerve growth-promoting factor, being salutary in encouraging the growth of axons in peripheral nerves [36]. Astragaloside IV, an active ingredient in RA, contributed to sciatic nerve regeneration and functional recovery in mice. The mechanism underlying this effect may be associated with the upregulation of growth-associated protein-43 expression [37]. RA extract promoted neural-directed differentiation of mesenchymal stem cells into nerve cells* in vitro *and also had neuroprotective effects on the central nervous system [38, 39].

This review identified a relatively large amount of evidence on the effectiveness of RA-based interventions, either tested alone or tested in combined remedies, for the prevention and treatment of OXAL induced peripheral neuropathy. Compared with no intervention or conventional western medicine, RA-based interventions have the potential of being more effective in relieving CIPN. RA-based interventions also lead to improvement of SNCV. No adverse event was reported and 2 trials reported Qol improvement after RA-based interventions. In China, there is a general perception that it could improve Qol for various conditions. However, clinical trials need to monitor and report Qol improvement.

Most of RA-based prescriptions included Danggui, Guizhi, Baishao, Jixueteng, Chuanxiong, and Honghua. These herbs may improve the effects of RA intervention on CIPN. Individualized treatment in TCM requires the modification of herbs with various symptoms in different patients. So the herbs included in RA-based prescriptions were heterogeneous. There were variations in the formulation, dosage, administration, and duration of treatment in the included trials. Even for herbal intervention of the same name, there were still differences in the specific composition or dose of included Chinese herbal medicine. Information about quality control was lacking on the development of the herbal preparations or the manufacture of herbal products. Future trials should provide information about standardization, including composition, quality control, and detailed regimens. The majority of trials compared RA-based intervention with no intervention; others used western conventional medicine as controls. Only 1 trial used a formal placebo control, so the positive effect should be interpreted conservatively.

This review has its limitations. We only included studies published in journals. Dissertations and conference papers were not included. Only high quality (improved Jadad score ≥ 4 points) trials were included. We excluded 38 trials with low quality or insufficient information for assessing risk of bias. Therefore, it may not be possible to achieve a complete summary of all existent evidence. Quantitative subgroup analysis exploring the effects of age, disease history, and duration could not be performed due to insufficient data. No multicenter, large-scale RCTs were identified. Most trials focused on short-term rather than long-term outcomes. Future trials should assure adequate concealment of allocation and blinding of outcome assessors.

## 5. Conclusions

From our study, we found that RA-based intervention may have clinical effectiveness for relieving OXAL induced peripheral neuropathy and lead to improvement of SNCV. However, the evidence is not sufficient. In the future, results from double-blind, multicenter, large-scale RCTs are needed to draw more definitive conclusions.

## Figures and Tables

**Figure 1:**
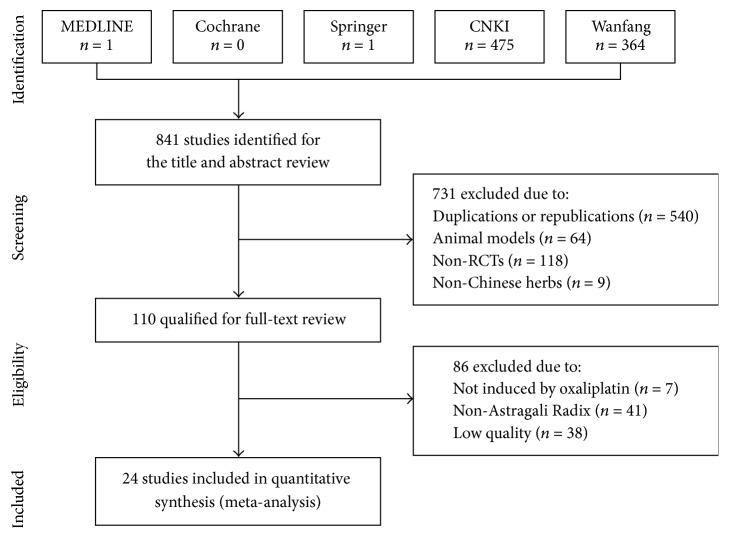
Flow chart of included studies in this systematic review.

**Figure 2:**
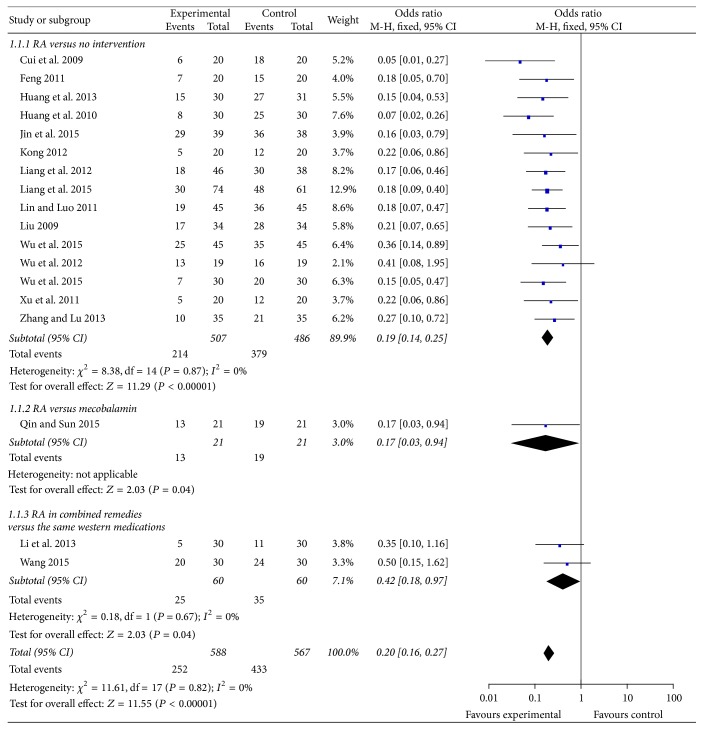
Forest plot of comparison: incidence of all-grade oxaliplatin-induced peripheral neuropathy.

**Figure 3:**
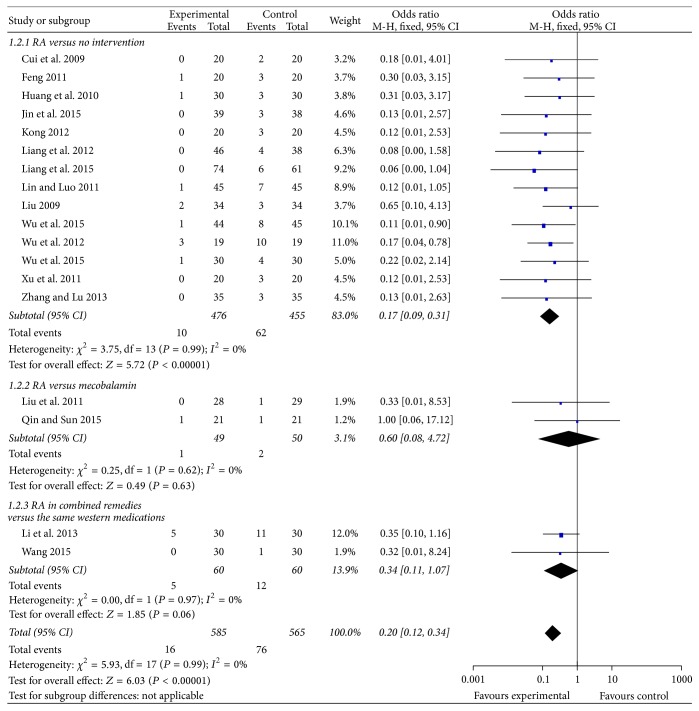
Forest plot of comparison: incidence of high-grade oxaliplatin-induced peripheral neuropathy.

**Figure 4:**
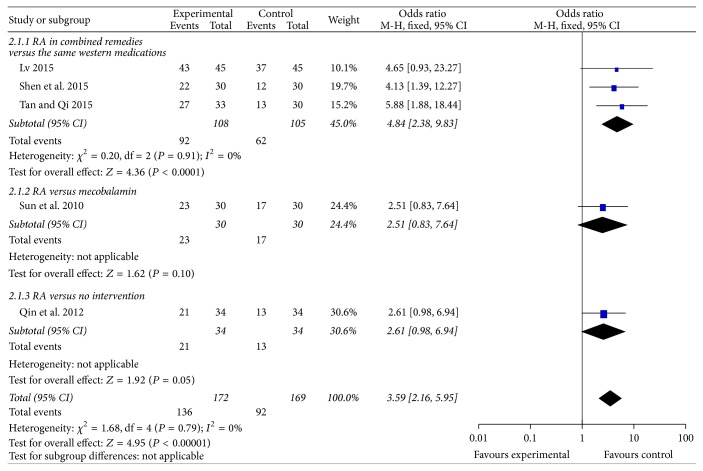
Forest plot of comparison: curative effects of Radix Astragali-based prescriptions on oxaliplatin-induced peripheral neuropathy.

**Figure 5:**
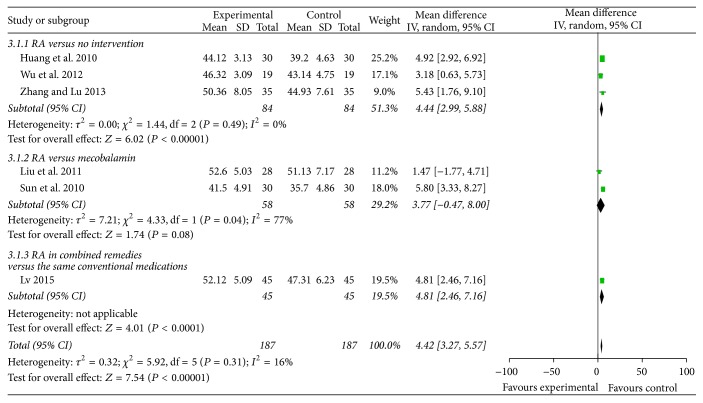
Forest plot of comparison: sensory nerve conduction velocity.

**Figure 6:**
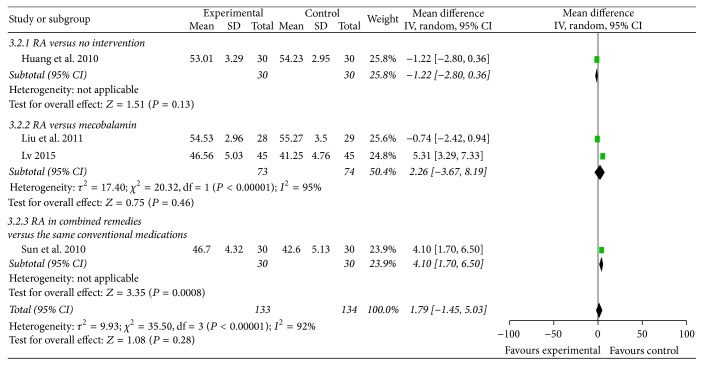
Forest plot of comparison: motor nerve conduction velocity.

**Table 1:** Grading scales used to evaluate oxaliplatin-induced peripheral neuropathy.

	Grade 1	Grade 2	Grade 3	Grade 4
Levi et al. [31]	Paresthesia or insensitive, complete relief in 1 week	Paresthesia or insensitive, complete relief in 14 days	Paresthesia or insensitive, complete relief in 21 days	Paresthesia or insensitive, combined with functional abnormality

Miller et al. [32]	Paresthesias and/or decreased tendon reflex	Severe paresthesia and/or mild anergia	Intolerable paresthesia and/or marked motor loss	Paralysis

CTCAE 4.03 [33]	Asymptomatic; loss of deep tendon reflexes or paresthesia	Moderate symptoms; limiting instrumental ADL	Severe symptoms; limiting self-care ADL	Life-threatening consequences; urgent intervention indicated

**Table 2:** Characteristics of studies included in this systematic review.

Author	Year	Sample size	Mean age (year) (median/range)	% men	Chemotherapy	Radix Astragali intervention	Control
Cui et al. [5]	2009	40	60	57.5	FOLFOX	Single herb extract	No intervention
Feng [6]	2011	40	28*～*55	67.5	FOLFOX	Compound prescription	No intervention
Huang et al. [7]	2013	61	62.3	62.3	FOLFOX	Compound prescription	No intervention
Huang et al. [8]	2010	60	46	71.7	FOLFOX	Compound prescription	No intervention
Jin et al. [9]	2015	77	47.2	48.1	FOLFOX	Compound prescription	No intervention
Kong [10]	2012	40	40*～*60	52.5	OXAL	Compound prescription	No intervention
Li et al. [11]	2013	60	50.1	31.7	FOLFOX	Compound prescription	GSH
Liang et al. [12]	2012	84	32*～*73	70.2	FOLFOX	Compound prescription	No intervention
Liang et al. [13]	2015	135	47.8	67.4	FOLFOX	Compound prescription	No intervention
Lin and Luo [14]	2011	90	51	54.4	FOLFOX	Compound prescription	No intervention
Liu et al. [15]	2011	60	61.5	64.3	FOLFOX, OXAL	Compound prescription	Mecobalamin
Liu [16]	2009	68	31*～*70	60.3	FOLFOX	Compound prescription	No intervention
Lv [17]	2015	90	53.0	53.3	OXAL	Compound prescription	Mecobalamin
Qin and Sun [18]	2015	42	55	61.9	FOLFOX	Compound prescription	Mecobalamin
Qin et al. [19]	2012	68	57.2	47.1	OXAL, TAX	Compound prescription	Cobamamide
Shen et al. [20]	2015	60	59.7	65.0	OXAL	Compound prescription	Mecobalamin
Sun et al. [21]	2010	60	55.3	63.3	FOLFOX, XELOX, TAX + 5-Fu	Compound prescription	Mecobalamin
Tan and Qi [22]	2015	63	31*～*70	60.3	OXAL	Compound prescription	Mecobalamin
Wang [23]	2015	60	52.3	50.0	FOLFOX	Compound prescription	Ca/Mg infusions
Wu et al. [24]	2015	89	49.2	70.8	Platinum, TAX,	Compound prescription	No intervention
Wu et al. [25]	2012	60	23*～*71	65.8	FOLFOX	Compound prescription	No intervention
Wu et al. [26]	2015	60	59.7	65.6	Platinum, TAX, VCR	Compound prescription	No intervention
Xu et al. [27]	2011	40	*∗*	45.0	OXAL	Compound prescription	No intervention
Zhang and Lu [28]	2013	70	31–77	65.7	FOLFOX	Single herb extract + thioctic acid	No intervention

*FOLFOX*: oxaliplatin + 5-fluorouracil + calcium folinate.

*5-Fu*: 5-fluorouracil.

*GSH*: glutathione.

*OXAL*: oxaliplatin.

*TAX*: taxol.

*VCR*: vincristine.

*XELOX*: oxaliplatin + capecitabine.

**Table 3:** Characteristics of Radix Astragali-based interventions.

Author	Year	Radix Astragali prescription	Administration	Course of treatment (d)	Dose (g)	Jadad score	CIPN grade	Curative effect	NCV	
Cui et al. [5]	2009	Huangqi Injection	i.v.	7	30 mL	4	+			5
Feng [6]	2011	Buyang Huanwu Decoction	ad us. ext	5 *∗* 6 chemotherapeutic cycles	120	4	+			6
Huang et al. [7]	2013	Huangqi Guizhi Wuwu Decoction	p.o.	5 *∗* 4 chemotherapeutic cycles	30	4	+			7
Huang et al. [8]	2010	Huangqi Guizhi Wuwu Decoction	ad us. ext	5 *∗* 4 chemotherapeutic cycles	100	4	+		+	8
Jin et al. [9]	2015	Self-made prescription	ad us. ext	10 *∗* 4 chemotherapeutic cycles	15	4	+			9
Kong [10]	2012	Self-made prescription	ad us. ext	6 *∗* 3 chemotherapeutic cycles	30	4	+			10
Li et al. [11]	2013	Self-made prescription	p.o.	5 *∗* 6 chemotherapeutic cycles	30	4	+			11
Liang et al. [12]	2012	Buyang Huanwu Decoction	p.o.	28	40	4	+			12
Liang et al. [13]	2015	Buyang Huanwu Decoction	p.o	28	30	4	+			13
Lin and Luo [14]	2011	Huangqi Guizhi Wuwu Decoction	ad us. ext	84	≥15	4	+			14
Liu et al. [15]	2011	Huangqi Guizhi Wuwu Decoction	p.o.	42	30	4	+		+	15
Liu [16]	2009	Self-made prescription	ad us. ext	14	≥15	4	+			16
Lv [17]	2015	Self-made prescription	ad us. ext	7	50	5		+	+	17
Qin and Sun [18]	2015	Self-made prescription	p.o.+ ad us. ext	14 *∗* 5 chemotherapeutic cycles	30	4	+			18
Qin et al. [19]	2012	Self-made prescription	ad us. ext	14	20	4		+		19
Shen et al. [20]	2015	Huangqi Guizhi Wuwu Decoction	ad us. ext	14	50	4		+		20
Sun et al. [21]	2010	Buyang Huanwu Decoction	ad us. ext	14	180	4		+	+	21
Tan and Qi [22]	2015	Self-made prescription	p.o.	10 *∗* 2 chemotherapeutic cycles	45	4		+		22
Wang [23]	2015	Huangqi Guizhi Wuwu Decoction	ad us. ext	3 *∗* 8 chemotherapeutic cycles	45	4	+			23
Wu et al. [24]	2015	Huangqi Guizhi Wuwu Decoction	p.o.	5 *∗* 4 chemotherapeutic cycles	30	4	+			23
Wu et al. [25]	2012	Buyang Huanwu Decoction	p.o.	112	60	4	+		+	25
Wu et al. [26]	2015	Self-made prescription	p.o.	7 *∗* 2 chemotherapeutic cycles	≥15	4	+			26
Xu et al. [27]	2011	Self-made prescription	ad us. ext	5 *∗* 2 chemotherapeutic cycles	30	4	+			27
Zhang and Lu [28]	2013	Huang qijing oral liquid	p.o.	5 *∗* 8 chemotherapeutic cycles	20	4	+		+	28

**Table 4:** Chinese herbs combination in Radix Astragali-based prescriptions.

Latin name	English name	Chinese name	Counts	Frequency (%)
*Angelica sinensis (Oliv.) Diels*	Radix Angelicae sinensis	Danggui	19	86.36
*Cinnamomum cassia Presl*	Ramulus Cinnamomi	Guizhi	17	77.27
*Paeonia lactiflora Pall.*	Radix Paeoniae Alba	Baishao	16	72.73
*Spatholobus suberectus Dunn*	Caulis spatholobi	Jixueteng	13	59.09
*Ligusticum chuanxiong Hort. *	Rhizoma Chuanxiong	Chuanxiong	12	54.55
*Carthamus tinctorius L.*	Flos Carthami	Honghua	10	50.00
*Prunus persica (L.) Batsch*	Semen persicae	Taoren	9	40.91
*Ziziphus jujuba Mill.*	Fructus Jujubae	Dazao	8	36.36
*Salvia miltiorrhiza Bge.*	Radix Salviae Miltiorrhizae	Danshen	7	31.82
*Zingiber officinale Rosc.*	Rhizoma Zingiberis (recens)	Jiang	7	31.82
*Pheretima aspergillum (E. Perrier)*	Pheretima	Dilong	7	31.82
